# Cancer and ageism

**DOI:** 10.3332/ecancer.2014.ed39

**Published:** 2014-07-03

**Authors:** Ulrich Wedding, Reinhard Stauder

**Affiliations:** 1 SIOG, Publication Committee Chair, Department of Internal Medicine II (Haematology, Oncology, Palliative Care), University Hospital Jena, Erlanger Allee 101 07747 Jena, Germany; 2 SIOG, Publication Committee Member, Department of Internal Medicine V (Haematology and Oncology), Innsbruck Medical University

The number of cancer cases being diagnosed is increasing. The single most important risk factor for the development of cancer is ageing. Improvements in cancer care over recent decades are substantial, however improvements in five year survival rates are less pronounced in elderly patients [1]. Do the association of cancer and ageing and the care for elderly cancer patients receive the attention they deserve?

In 1999 Laura Hutschins *et al*. published an analysis of the relative frequency of patients aged 65 years and older included into trials of the South-West-Oncology-Group (SWOG) and compared the data to the relative frequency of persons aged 65 years and older in the general US population. All in all elderly cancer patients are often excluded from clinical trials [2, 3]. Barriers are the in- and exclusion criteria, the referral of elderly patient to centres offering trials compared to younger patients is poorer, and participation offered less frequently, even if a trial is available [4]. Elderly specific trials improve the recruitment of elderly patients [5].

Together with the European Organization for Research and Treatment of Cancer (EORTC) and the America Study Alliance, the International Society of Geriatric Oncology (SIOG) recently suggested strategies to overcome the deficits of clinical research for elderly patients [6]. Clinical research to improve the care of elderly patients is more difficult than for younger ones. Elderly patients suffer more often from multimorbidity with the cancer diagnosis being just one disease among others, they more often face limitations in functional status as well as experiencing a greater incidence of depression, cognitive impairment and social isolation. In addition the aging process is very heterogeneous. Whereas some elderly patients are very fit, others are compromised or frail [7]. This heterogeneous process of aging is well known in geriatric medicine and led to the establishing of a geriatric assessment to describe the patient`s deficits and resources and to develop an individual treatment plan. So far the transfer of geriatric assessment into oncological care could demonstrate that it identifies deficits of patients missed before and that these restrictions are associated with toxicity and reduced quality of life and clinical outcome. Integration of geriatric assessment can result in a modification of the treatment plan [8].

All in all elderly cancer patients deserve a personalized approach to medicine, reflecting the overall health and social situation, to define the individual aims of treatment.

Why do they remain so poorly addressed in clinical cancer research? One reason is that clinical research in this group of patients is more difficult than in younger ones, however, should we not address topics just because they are difficult? Another reason is the poor image of geriatric care in general. A Norwegian study asked medical students, general practitioners, and consultants for the image of 26 different specialities of medicine - geriatric medicine was at the bottom line in all three groups. Thus the image of the care for older patients is poor, irrespective of the setting of care (general practice vs. hospital) and the amount of experience in the field (students vs. general practitioners and consultants) [9].

The first step to overcome ageism in cancer care is to accept that the topic has been neglected in the past for many different reasons. The second step is to develop strategies to address the specialised needs of elderly cancer patients and to overcome ageism. The patients deserve these steps.

SIOG (http://www.siog.org/) addresses the special situation of elderly cancer patients within its yearly conferences, this years conference will be held in Lisbon October 2014 (http://www.siog.org/index.php?option=com_content&view=article&id=278&Itemid=177), in several publications covering important topics (http://www.siog.org/index.php?option=com_content&view=article&id=246&Itemid=92), in the monthly edition of the Journal of Geriatric Oncology (http://www.geriatriconcology.net/) and among others in videos talks (http://ecancer.org/advanced-search/video.php?_limit=15).
